# Whole Soy Flour Incorporated into a Muffin and Consumed at 2 Doses of Soy Protein Does Not Lower LDL Cholesterol in a Randomized, Double-Blind Controlled Trial of Hypercholesterolemic Adults^1^,^2^

**DOI:** 10.3945/jn.115.219873

**Published:** 2015-10-07

**Authors:** Emily MT Padhi, Heather J Blewett, Alison M Duncan, Randolph P Guzman, Aileen Hawke, Koushik Seetharaman, Rong Tsao, Thomas MS Wolever, D Dan Ramdath

**Affiliations:** 3 Guelph Food Research Centre, Agriculture and Agri-Food Canada, Guelph, Canada;; 4 Department of Human Health and Nutritional Sciences, University of Guelph, Guelph, Canada;; 5 Canadian Centre for Agri-Food Research in Health and Medicine, Agriculture and Agri-Food Canada, Winnipeg, Canada; and; 6 Glycemic Index Laboratories Inc., Toronto, Canada

**Keywords:** cardiovascular disease, dose-response, isoflavones, LDL cholesterol, randomized clinical trial, soy flour

## Abstract

**Background:** Soy protein may reduce coronary heart disease (CHD) risk by lowering LDL cholesterol, but few studies have assessed whether whole soy flour displays a similar effect.

**Objective:** The aim of this study was to assess the dose effect of whole soy flour incorporated into muffins on plasma LDL cholesterol in hypercholesterolemic adults.

**Methods:** Adults aged 30–70 y (*n* = 243) with elevated LDL cholesterol (≥3.0 and ≤5.0 mmol/L) were stratified by LDL cholesterol and randomly assigned to consume 2 soy muffins containing 25 g soy protein [high-dose soy (HDS)], 1 soy and 1 wheat muffin containing 12.5 g soy protein and 12.5 g whey protein [low-dose soy (LDS)], or 2 wheat muffins containing 25 g whey protein (control) daily for 6 wk while consuming a self-selected diet. Fasting blood samples were collected at weeks 0, 3, and 6 for analysis of plasma lipids [total, LDL, and HDL cholesterol and triglycerides (TGs)], glucose, insulin, C-reactive protein (CRP), and isoflavones. Blood pressures also were measured. Dietary intake was assessed at weeks 0 and 4 with the use of 3 d food records. Treatment effects were assessed with the use of intention-to-treat analysis with multiple imputation and LDL cholesterol as the primary outcome.

**Results:** In total, 213 (87.6%) participants completed the trial. Participants were primarily Caucasian (83%) and mostly female (63%), with a mean ± SD body mass index (in kg/m^2^) of 28.0 ± 4.6 and systolic and diastolic blood pressures of 122 ± 16 and 77 ± 11 mm Hg, respectively. Despite a dose-dependent increase in plasma isoflavones (*P* < 0.001), neither HDS nor LDS had a significant effect on LDL cholesterol compared with control (mean ± SEM changes: control, −0.04 ± 0.05 mmol/L; HDS, 0.01 ± 0.05 mmol/L; and LDS, −0.04 ± 0.06 mmol/L). There were no significant treatment effects on total or HDL cholesterol, TGs, CRP, homeostatic model assessment of insulin resistance, blood pressure, or the Framingham 10-y CHD risk score.

**Conclusion:** Consuming 12.5 or 25 g protein from defatted soy flour incorporated into muffins does not reduce LDL cholesterol or other CHD risk factors in hypercholesterolemic adults. This trial was registered at clinicaltrials.gov as NCT01547585.

## Introduction

Coronary heart disease (CHD)^8^ is a leading cause of death worldwide (1, 2), and it is well established that reducing plasma LDL cholesterol reduces the incidence of CHD (3). Improving dietary quality is recommended for cardiovascular health promotion (4), and increased consumption of plant-based foods may lower CHD risk by modulating circulating plasma lipids (5–7). Soybeans (*Glycine max*) have received considerable attention, with evidence from both epidemiologic and interventional studies suggesting that soy-based diets reduce LDL cholesterol (7–10). An early meta-analysis concluded that soy protein lowered LDL cholesterol by 12.9% (8), and, in 1999, the FDA approved a food labeling health claim associating a daily intake of 25 g soy protein with reduced CHD risk (11). However, subsequent meta-analyses showed that the LDL cholesterol reduction by soy protein was more modest, between 4% and 6% (12–19). Many ensuing studies have provided contradictory findings on the LDL cholesterol–lowering effect of soy; this continues to create disagreements in the regulatory and scientific community (20–23).

In 2010, the European Food Safety Authority rejected a health claim petition for soy protein and LDL cholesterol citing lack of evidence for a cause-and-effect relation for soy protein, specifically (22). However, in a recent assessment by Health Canada, it was concluded that interventions with isolated soy protein (ISP) and soy protein concentrate resulted in a significant lowering of LDL cholesterol in 33% of studies examined (23). Additionally, a meta-analysis performed by Health Canada concluded that soy significantly reduces LDL cholesterol, and an industry petition for a soy health claim in Canada has been approved (24).

A potential source of inconsistent findings is that although soy contains several bioactive components that may reduce LDL cholesterol, it is not clear which of these is responsible. Furthermore, variations in soy products used in intervention studies have made interpretation of the results difficult. It is therefore important to evaluate the LDL cholesterol–lowering effect of soy in different food matrices. Most trials have focused on ISP and isoflavones (10); however, defatted whole soy flour (DWSF), used in large amounts in the food industry, contains several potentially active components and has not been adequately assessed for its effect on LDL cholesterol. Moreover, few studies have examined the dose-response relation between soy protein intake and LDL cholesterol; this is required to establish the minimum effective dose of soy and it is essential to implementing regulatory guidelines.

Therefore, the aim of this study was to determine the dose effect of DWSF incorporated into a baked food product on LDL cholesterol in healthy adults with hypercholesterolemia. We hypothesized that DWSF would elicit a dose-dependent reduction in LDL cholesterol.

## Methods

### 

#### Participants.

Men and nonpregnant women with elevated LDL cholesterol (≥3.0 and ≤5.0 mmol/L) and not taking cholesterol-lowering medications were recruited from 3 Canadian cities—Guelph, Toronto, and Winnipeg—and the surrounding areas between May 2012 and September 2013 with the following inclusion criteria: aged 30–70 y; BMI (in kg/m^2^) ≥18.5 and ≤40.0 with stable weight (i.e., weight change of ≤3 kg in 3 mo before study enrollment); and not on prescribed or nonprescribed medications or herbal or nutritional supplements known to affect blood lipids, except for stable doses of thyroxine, oral contraceptive agents, hormone replacement therapy, and medications for controlling blood pressure. The following exclusion criteria were also applied: users of prescribed lipid-lowering medications (e.g., statins); smoking ≥1 cigarette/d; alcohol intake >2 drinks (28 g ethanol)/d; unstable body weight or intention to lose or gain weight; regular consumption of soy, defined as ≥5 servings of soy/wk where 1 serving of soy was a soy-based food or supplement containing at least 6.25 g of soy protein per serving (11); the presence of any known food allergies; concurrent participation in other scientific studies; unwillingness to consume a soy or wheat baked product; saturated fat intake ≥15% of total energy intake as determined by a 3 d food record obtained before study enrollment; any major surgical or medical event within the previous 3 mo; presence of a gastrointestinal disorder or medication that alters the digestion and absorption of nutrients; fasting plasma TGs ≥4.0 mmol/L; abnormal kidney and liver function assessed as plasma aspartate transaminase and plasma alanine transaminase ≥1.5 times the upper limit of normal; abnormal kidney function assessed by plasma urea and creatinine ≥1.8 times the upper limit of normal; presence of diabetes mellitus, defined as fasting plasma glucose ≥7.0 mmol/L (25); or use of insulin or any oral hypoglycemic agents. Individuals who reported recent use of antibiotics (≤6 wk before study enrollment) were asked to undergo a waiting period before enrolling in the study.

#### Screening.

**FIGURE 1:**
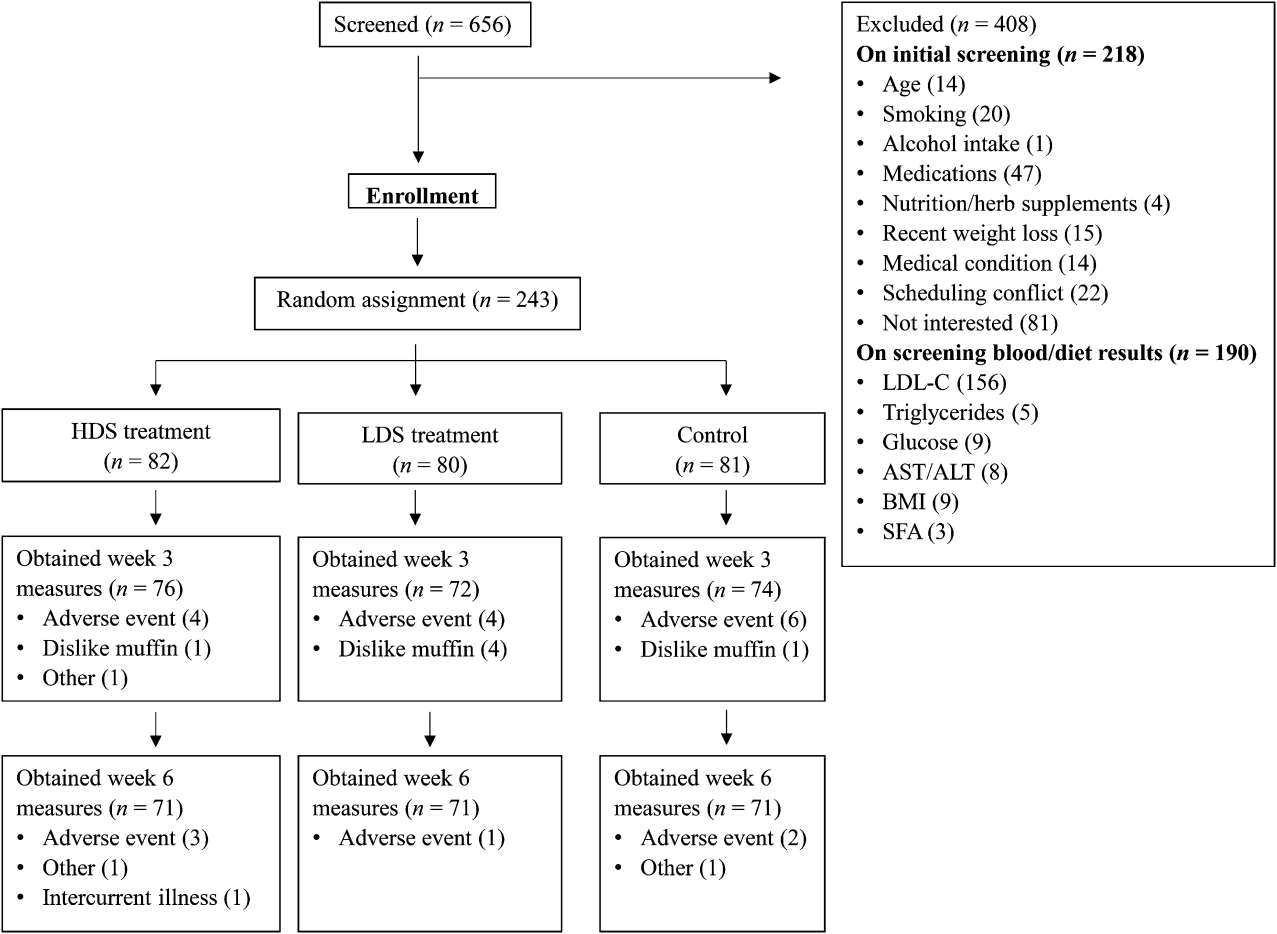
Consolidated Standards of Reporting Trials diagram showing flow of participants through trial. The HDS group received 25 g/d soy protein; the LDS group received 12.5 g/d soy protein and 12.5 g/d whey protein; and the control group received 25 g/d whey protein. ALT, alanine transaminase; AST, aspartate transaminase; HDS, high-dose soy; LDL-C, LDL cholesterol; LDS, low-dose soy.

Participants were recruited locally with the use of flyers, radio and newspaper advertisements, and Internet postings. A summary of participant recruitment, enrollment, completion, and attrition is presented in **Figure 1**. All study procedures were reviewed and approved by the ethics review boards of the respective clinical trial centers, and all participants provided signed informed consent after receiving verbal and written information about the study. At each trial site, interested persons underwent an initial telephone screening, and eligible persons were invited to visit the clinical trial center for a study orientation and screening interview. Participants then provided a fasting blood sample, had height, weight and blood pressure measured, and were instructed on the completion of a 3 d food record, which was returned 1 wk later and analyzed centrally by a trained study assistant to determine study eligibility. Screening blood samples were analyzed by LifeLabs Medical Laboratory Services, St. Michael’s Hospital, and the Canadian Centre for Agri-Food Research in Health and Medicine.

#### Random assignment and concealment.

Eligible participants were stratified according to LDL cholesterol at screening (low stratum = LDL cholesterol ≥3.0 mmol/L and ≤3.8 mmol/L; high stratum = LDL cholesterol >3.8 mmol/L and ≤5.0 mmol/L), then randomly assigned to 1 of 3 study groups at their baseline visit. Treatment assignments were provided in code (A, B, or C) in sealed, sequentially numbered opaque envelopes prepared by the study statistician. Assignment was done by random number generated by the study statistician with provision for 200 participants (100 in each LDL cholesterol stratum) at each study site. The treatment groups included a high-dose soy (HDS) group that received 2 soy muffins daily (containing a total of 25 g soy protein); a low-dose soy (LDS) group that received 1 soy and 1 wheat muffin daily (containing 12.5 g soy protein and 12.5 g whey protein, respectively); and a control group that received 2 wheat muffins daily (containing a total of 25 g whey protein). All study staff except for the study statistician were blind to the treatment group assignment.

#### Intervention.

The intervention comprised muffins made from soy or wheat flour that were developed and produced at the Bake Lab, Department of Food Science, University of Guelph. The muffins were isocaloric, matched for macronutrient content, and designed to contain a minimum of 12.5 g soy protein (∼27 g DWSF per muffin) so that 2 muffins would deliver 25 g soy protein daily, which meets the US FDA recommended intake of soy protein for reduced CHD risk (11). The study muffins were devoid of ingredients known to influence circulating lipids, such as plant sterols. Insoluble fiber (WC150 wheat fiber; CreaFill Fibers) was added to the wheat muffin to increase fiber content. DWSF (Cargill) was provided as an in-kind contribution by Soy 20/20. Commercial soft wheat flour was donated by Kraft Canada. Whey isolate INPRO 90 protein powder was from Vitalus Nutrition. For treatment blinding and to mask differences in taste, an underlying artificial vanilla flavoring was used, followed by either lemon or banana flavoring (David Michael & Co.). Muffins were produced and packaged weekly, stored at −22°C at the University of Guelph, and delivered frozen to participating study centers. A random selection of muffins from each production batch was stored for proximate analysis (Maxaam Analytics) (**Table 1**). In addition, for every new batch of flour used, samples of flour and muffins were analyzed commercially for microbial and toxin content. The muffins were packaged in translucent plastic sleeves with the use of a 16 inch Impulse Sealer with Cutter (ULINE). A general label containing an arbitrary barcode and a discreet code (containing assignment, muffin production batch, and date of preparation) was affixed to each plastic sleeve. Each sleeve contained either 2 soy muffins, 1 soy muffin and 1 wheat muffin, or 2 wheat muffins.

**TABLE 1:** Nutrient composition of intervention soy muffin and control wheat muffin^1^

	Weight,^2^ g	Energy, kcal/100 g FW	Protein,^3^ g/100 g FW	Fat, g/100 g FW	Carbohydrate, g/100 g FW	Ash, g/100 g FW	Moisture, g/100 g FW	Fiber, g/100 g FW	Isoflavones,^4^ mg/g DW
Soy muffin	97 ± 2	335 ± 10.2	14.3 ± 1.2	13.7 ± 1.1	39.5 ± 4.5	2.3 ± 0.2	30.2 ± 2.4	5.2 ± 0.4	0.61 ± 0.1
Wheat muffin	88 ± 2	384 ± 4.2	14.8 ± 1.7	16.4 ± 0.1	45.2 ± 1.2	0.9 ± 0.0	23.4 ± 0.9	1.5 ± 0.2	ND

1 Values are means ± SEMs of triplicate analysis. Values obtained with the use of muffins collected on different production days (soy, *n* = 18; wheat, *n* = 15). Analysis was by AOAC methodology (protein, AOAC 992.15; ash, AOAC 923.03; fat, by gravimetric analysis with the use of acid hydrolysis, AOAC 922.06, 933.05; total dietary fiber, AOAC 991.43, 985.29). Carbohydrates were calculated by difference. DW, dry weight; DWSF, defatted whole soy flour; FW, fresh weight; ND, not detected.

2 Mean ± SEM weight per muffin as eaten.

3 Soy muffins contained protein primarily from DWSF; a small portion of protein (∼1.5 g) was contributed by wheat flour.

4 Reported as isoflavone aglycone equivalents/g DW.

#### Study procedures and measurements.

We conducted a 6 wk double-blind, multicenter, randomized, controlled clinical trial with a parallel-group design, as outlined in Figure 1. Participants were asked to maintain their usual diet, avoid sources of soy external to the study muffins, and maintain regular physical activity routines throughout the study. Study muffins were provided weekly to participants, who were instructed to consume the muffins in place of breakfast or as a snack, according to their preference (e.g., defrosted and consumed cold, heated, toasted, and/or with spreads and beverages), and could consume both muffins at the same time or separately during different meals. Participants were also asked to report on any adverse effects and if any major changes to diet or routine were experienced in the previous week. For study visits, participants were asked to avoid alcohol for 24 h and over-the-counter medications for 48 h, and limit strenuous physical activity on the evening before their study visit. They arrived at each trial site in the morning after a 10–12 h overnight fast. A fasting blood sample (15 mL) was obtained at weeks 0, 3, and 6 for the analysis of plasma lipids, C-reactive protein (CRP), glucose, insulin, and soy isoflavones. In addition, body weight and height were measured with scales and stadiometers approved for use in clinical settings, and seated blood pressure was measured after a 5 min rest (reported as the average of 3 measures). Waist circumference was measured at baseline and at the final study visit (26, 27).

Fasting blood samples were collected into spray-coated lithium heparin BD evacuated tubes and centrifuged (1300 × *g*; 10 min; 21°C) within 10 min of collection to obtain plasma. Aliquots of plasma were transferred into 2.0 mL capped Sarstedt microtubes and stored at −80°C until analysis. Plasma lipids, high-sensitivity CRP, and glucose were analyzed centrally with the use of a Cobas c 111 clinical chemistry analyzer (Roche Diagnostics) by enzymatic colorimetric, particle-enhanced turbidimetric, and UV-based methods, respectively, at the Canadian Centre for Agri-Food Research in Health and Medicine. In this system, LDL cholesterol was measured directly and the total-to-HDL cholesterol ratio was derived. Insulin was measured centrally with the use of an electrochemiluminescence immunoassay on a Roche E modular E170 immunoassay analyzer at Unicity Laboratory Services. Insulin resistance (HOMA-IR) was derived with the use of the Homeostasis Model Assessment 2 online calculator (28). The Framingham risk assessment tool for adults (≥20 y) without heart disease or diabetes was used to assess 10 y CHD risk with the use of the following variables: age, gender, total cholesterol, HDL cholesterol, smoking status, systolic blood pressure, and treatment for hypertension (29). In cases in which participant values fell outside the range of admissible values defined by the risk assessment tool (e.g., values for HDL cholesterol must fall between 20 mg/dL (0.52 mmol/L) and 100 mg/dL (2.59 mmol/L), the maximum or minimum value was used in the calculator.

All results were entered onto case report forms (CRFs) that were sent by facsimile to the coordinating center (Guelph Food Research Centre, Agriculture and Agri-Food Canada). Blood results were tabulated and sent from the central laboratory via secured e-mail. All CRF data were verified and validated via double entry.

Assessment of dietary intake was achieved through 3 d food records collected at baseline and during week 4 of the study. Participants were instructed to record the quantity of all foods and caloric beverages consumed over 2 typical weekdays (nonconsecutive and consecutive recording of weekdays was permitted) and 1 weekend day. Completed records were verified by a trained study assistant and then analyzed with the use of ESHA Food Processor (SQL Nutrition Analysis Software, version 10.1.1, 2007) at the coordinating site. All food record entries were verified 2 times.

An aliquot of plasma was transported from participating clinical trial centers to the Guelph Food Research Centre for isoflavone analysis with the use of an established LC tandem MS method (30, 31). Briefly, thawed samples were treated with β-glucuronidase type H1 from *Helix pomatia* (Sigma-Aldrich), followed by protein precipitation with the use of acidified methanol, filtration, and analysis via LC tandem MS (32). An equol producer was defined as a participant with plasma equol concentrations >20 nmol/L (33). Currently, there are no available guidelines for the classification of an *O*-desmethylangolensin producer. Therefore, for the purpose of this study, an *O*-desmethylangolensin producer was considered to be anyone who produced detectable amounts of this metabolite in his or her plasma.

#### Assessment of compliance.

At weekly study visits, participants were asked to return empty muffin packaging sleeves or uneaten muffins, and to report on the number of muffins consumed during the previous week. If a participant failed to bring in their empty muffin packaging, they were verbally questioned about whether they were able to consume all the study muffins during the course of the previous week. Additionally, compliance was assessed through the quantification of plasma isoflavones at weeks 0, 3, and 6.

#### Sample size.

The required sample size calculation was based on previous meta-analyses (12–18), a trial with a similar design and outcome measures that examined the cholesterol-lowering effect of oat β-glucan (34), and a biologically significant reduction in LDL cholesterol. We calculated that in order to detect a 7% (0.28 mmol/L) reduction in baseline LDL cholesterol in the HDS group, with an 80% power and SD of 0.64 mmol/L (34), 72 participants were required for each treatment arm. After we adjusted for an expected 12% dropout rate, the total number of participants required was 243.

#### Statistical analysis.

Initial statistical analysis was performed by a study statistician who was unaware of the treatment assignments. All statistical analyses were performed with the use of SAS version 9.4 with a 2-tailed type I error rate of 0.05. Data are expressed as means ± SEMs unless otherwise stated. Outliers were identified from the calculated IQR; values >3 times the IQR (strong outlier) were examined and removed if justified after consultation with the CRF and source data, and discussions with study coordinators at local sites. In total, there were 9936 values at all time points (i.e., combined measurements at weeks 0, 3, and 6) from which 115 outliers were identified and 54 were removed before analysis; no LDL cholesterol values were removed as outliers. Data removed at baseline were replaced with group mean values. Missing data at weeks 3 and 6 were replaced by multiple imputation (PROC MI) before analysis, as described by Schafer (35). The latter involved generating 5 complete data sets with the use of regression estimates obtained from observed values, after which mean values of the imputed data were inserted for the missing values. Data were checked for normality with the use of the Shapiro-Wilk test (PROC UNIVARIATE), and variables not normally distributed were transformed with the use of ln (tTGs, insulin, and HOMA-IR) and reciprocal transformation (CRP). A 1-factor ANOVA (PROC GLM) was used to assess group differences at baseline.

The change (week 6 minus baseline) in LDL cholesterol, the primary outcome, was analyzed by intention-to-treat with the use of the multiple imputation method for missing data (PROC MI), as outlined above, and followed the fully conditional specification approach (36). A 1-factor ANOVA was used to evaluate the effect of treatment on change in LDL cholesterol, as well as secondary outcomes, including total cholesterol, HDL cholesterol, TGs, total-to-HDL cholesterol ratio, systolic and diastolic blood pressure, glucose, and insulin (PROC GLM). A Kruskall-Wallis test was used to compare the effect of treatment on changes in: CRP; HOMA-IR; Framingham risk score; median plasma isoflavone concentrations between groups at weeks 3 and 6; and change in LDL cholesterol among equol producers and nonproducers (PROC NPAR1WAY). Paired *t* tests were used to compare differences in dietary intake at baseline (week 0) and midpoint (week 4) (PROC TTEST). ANCOVA was performed on primary and secondary outcomes variables to ascertain the effects of age, gender, BMI, center, total energy, macronutrients, and dietary fiber (PROC MIXED). Repeated-measures ANOVA was used to assess change from baseline in primary and secondary outcome variables by treatment at the study time points (PROC MIXED). Independent *t* tests were used to compare the net change in primary and secondary outcome variables in pooled soy treatments compared with control (PROC TTEST).

## Results

At the end of the trial, 213 (87.6%) of the original 243 participants completed the study, as outlined in Figure 1. During the study, 129 participants experienced at least one adverse effect; however, none was reported as a serious adverse event. In total, 30 participants dropped out for the following reasons: adverse event related or unrelated to the study product, (*n* = 20); dislike of the study product (*n* = 6); death in the family (*n* = 2); concern about soy intake and breast cancer risk (*n* = 1), and recurrence of cancer unrelated to the study (*n* = 1). The number of participants discontinuing participation was similar across treatment arms (HDS, *n* = 11; LDS, *n* = 9; control, *n* = 10), and the majority dropped out during the first half of the trial (i.e., 22 of 30). A summary of participant baseline demographics, medical conditions, and use of medications is shown in **Table 2**. Study participants had a mean ± SD age of 55.0 ± 8.8 y and the majority were women (63.4%). Participants were mostly Caucasian (82.7%), followed by South Asian (6.6%), Southeast Asian (2.5%), East Asian (2.5%), black (2.5%), Arab/West Asian (0.8%), Aboriginal (0.4%), and mixed ethnicity (2.1%; one each of black/Caucasian, Hispanic/Caucasian, Aboriginal/Caucasian, and black/South Asian).

**TABLE 2:** Baseline demographic, clinical, and anthropometric characteristics of hypercholesterolemic adults in a soy dietary intervention trial and allocated to HDS, LDS, and control groups^1^

Variables	All (*n* = 243)	HDS group (*n* = 82)	LDS group (*n* = 80)	Control group (*n* = 81)
Age, y	55.0 ± 8.8	56.0 ± 9.2	55.0 ± 9.0	54.0 ± 8.2
BMI, kg/m^2^	28.0 ± 4.6	27.7 ± 4.4	28.1 ± 4.9	28.1 ± 4.4
Waist circumference, cm	94.8 ± 11.5	93.1 ± 10.1	95.3 ± 12.5	95.9 ± 11.7
Systolic BP, mm Hg	122 ± 16	118 ± 16	123 ± 16	123 ± 17
Diastolic BP, mm Hg	77 ± 11	75 ± 12	78 ± 10	78 ± 9
Male/Female, *n*/*n*	89/154	29/53	29/51	31/50
Medical conditions				
Cardiovascular^2^	43	9	18	16
Endocrine^3^	21	8	7	6
Neurological^4^	25	7	10	8
Gastrointestinal^5^	16	8	5	3
Asthma	7	3	2	2
Musculoskeletal^6^	26	11	6	9
Dermatologic^7^	9	4	3	2
Other^8^	12	3	4	5
Prescribed medications	110	32	35	43
Over-the-counter medications	79	15	22	42
Nutritional supplements	144	53	48	43
Herbal supplements	23	11	3	9

1 Values are means ± SDs or *n*, unless otherwise indicated. The HDS group received 25 g/d soy protein; the LDS group received 12.5 g/d soy protein and 12.5 g/d whey protein; and the control group received 25 g/d whey protein. BP, blood pressure; HDS, high-dose soy; LDS, low-dose soy.

2 Includes hypertension, heart murmur, and Brugada syndrome.

3 Includes hypo- and hyperthyroidism, Grave disease, hot flashes, and prediabetes.

4 Includes depression, anxiety, bipolar disorder, attention deficit hyperactivity disorder, insomnia, narcolepsy, migraines, atypical neurofibromatosis, mild cognitive impairment, and sleep apnea.

5 Includes gastroesophageal reflux disorder, acid reflux, indigestion, Crohn disease, constipation, gastric ulcer, and irritable bowel syndrome.

6 Includes arthritis, back and joint pain, osteoporosis, shingles, and restless leg syndrome.

7 Includes psoriasis, rash, hives, eczema, and rosacea.

8 Includes glaucoma, hormone replacement therapy, prostate hyperplasia, acne, hepatitis B, iron deficiency, allergies, lung cancer (remission), kidney stones, ovarian cancer (remission), uterine cancer (remission), polio, breast cancer (remission), and alopecia.

Over the study period, compliance, as evaluated by uneaten muffins, was 98.8%, 99.0%, and 98.7% within the HDS, LDS, and control treatments, respectively. Overall, macronutrient intake at baseline and week 4 was not significantly different (**Table 3**). However, among the treatment groups, energy intake significantly increased during the study period by 226 ± 57 kcal/d in the HDS group, 267 ± 58 kcal/d in the LDS group, and 319 ± 53 kcal/d in the control group (*P* < 0.001). Protein intake was not significantly different from baseline, but intake of fat, saturated fat, and total carbohydrate as a percentage of total energy and energy-adjusted dietary fiber increased (*P* < 0.05) in all groups. Cholesterol intake significantly decreased in the HDS (−42 ± 18 mg/d) and LDS (−30 ± 15 mg/d) groups, but not in the control group (*P* < 0.05).

**TABLE 3:** Energy and nutrient intakes at baseline (week 0) and midpoint (week 4) in the HDS, LDS, and control groups^1^

	HDS group		LDS group		Control group	
	Baseline (*n* = 82)	Midpoint (*n* = 72)	Baseline (*n* = 80)	Midpoint (*n* = 72)	Baseline (*n* = 81)	Midpoint (*n* = 73)
Energy, kcal/d	1960 ± 60	2180 ± 60*	1930 ± 70	2200 ± 60*	1920 ± 60	2270 ± 70*
Protein, % energy	17.8 ± 0.4	18.3 ± 0.3	17.7 ± 0.5	17.9 ± 0.4	17.2 ± 0.4	17.6 ± 0.4
Carbohydrate, % energy	51.8 ± 1.0	47.5 ± 0.7*	52.7 ± 0.8	49.1 ± 0.8*	51.9 ± 0.9	48.9 ± 0.7*
Total fat, % energy	31.4 ± 0.7	33.7 ± 0.5*	30.2 ± 0.6	32.6 ± 0.6*	32.1 ± 0.7	33.5 ± 0.6 *
Saturated fat, % energy	9.9 ± 0.3	8.0 ± 0.3*	9.3 ± 0.3	7.7 ± 0.3*	10.0 ± 0.3	8.2 ± 0.3*
Total dietary fiber, g/1000 kcal	12.7 ± 0.4	11.8 ± 0.3^a,^*	11.8 ± 0.5	10.6 ± 0.4^a^*	11.8 ± 0.5	8.8 ± 0.4^b,^*
Cholesterol, mg/d	260 ± 15	210 ± 12^a,^*	267 ± 16	228 ± 14^a^*	253 ± 14	278 ± 15^b^
Sodium, mg/d	2570 ± 96	2470 ± 98	2580 ± 126	2510 ± 91	2680 ± 110	2580 ± 110

1 Values are means ± SEMs based on data obtained from 3 d food records. The HDS group received 25 g/d soy protein; the LDS group received 12.5 g/d soy protein and 12.5 g/d whey protein; and the control group received 25 g/d whey protein. *Significantly different from baseline, *P* < 0.05 (paired *t* tests). Labeled midpoint (week 4) means in a row without a common letter differ, *P* < 0.05 (1-factor ANOVA). HDS, high-dose soy; LDS, low-dose soy.

At baseline, there were no significant group differences in BMI, waist circumference, diastolic blood pressure, plasma lipids, glucose, insulin, HOMA-IR, and CRP (Table 3 and **Table 4**); lower systolic blood pressure in the HDS group approached significance (*P* = 0.050). At the end of the study period, there were no significant differences in the respective pooled means ± SEMs for BMI (28.0 ± 0.9), waist circumference (95.2 ± 2.2 cm), systolic (121 ± 3 mm Hg) or diastolic (76 ± 2 mmHg) blood pressure.

**TABLE 4:** Absolute data and net and percentage changes in plasma lipids, HOMA-IR, and CRP in the total study population and the HDS, LDS, and control groups^1^

	All (*n* = 243)	HDS group (*n* = 82)	LDS group (*n* = 80)	Control group (*n* = 81)
Total cholesterol, mmol/L				
Week 0	5.72 ± 0.05	5.65 ± 0.09	5.73 ± 0.09	5.77 ± 0.08
Week 3	5.56 ± 0.04	5.46 ± 0.08	5.64 ± 0.08	5.59 ± 0.08
Week 6	5.57 ± 0.04	5.56 ± 0.08	5.56 ± 0.07	5.60 ± 0.08
Net change	−0.14 ± 0.03	−0.10 ± 0.05	−0.17 ± 0.06	−0.16 ± 0.04
% change	−2.0 ± 0.5	−1.2 ± 0.9	−2.3 ± 1.1	−2.6 ± 0.7
LDL cholesterol, mmol/L				
Week 0	4.06 ± 0.05	4.01 ± 0.08	4.02 ± 0.08	4.15 ± 0.08
Week 3	4.03 ± 0.04	3.93 ± 0.08	4.06 ± 0.07	4.09 ± 0.07
Week 6	4.03 ± 0.04	4.02 ± 0.08	3.98 ± 0.07	4.10 ± 0.08
Net change	−0.02 ± 0.03	0.01 ± 0.05	−0.04 ± 0.06	−0.04 ± 0.05
% change	0.2 ± 0.7	1.0 ± 1.2	0.0 ± 1.4	−0.3 ± 1.2
HDL cholesterol, mmol/L				
Week 0	1.51 ± 0.03	1.51 ± 0.04	1.54 ± 0.05	1.47 ± 0.04
Week 3	1.51 ± 0.03	1.50 ± 0.04	1.55 ± 0.05	1.48 ± 0.04
Week 6	1.52 ± 0.03	1.53 ± 0.05	1.54 ± 0.05	1.48 ± 0.04
Net change	0.01 ± 0.01	0.02 ± 0.02	0.00 ± 0.02	0.00 ± 0.01
% change	1.2 ± 0.7	1.8 ± 1.1	0.8 ± 1.5	1.0 ± 1.0
Total:HDL cholesterol ratio				
Week 0	4.03 ± 0.07	3.93 ± 0.11	3.98 ± 0.12	4.19 ± 0.13
Week 3	3.92 ± 0.07	3.85 ± 0.11	3.91 ± 0.13	4.01 ± 0.12
Week 6	3.91 ± 0.07	3.83 ± 0.11	3.87 ± 0.12	4.04 ± 0.13
Net change	−0.12 ± 0.03	−0.10 ± 0.04	−0.11 ± 0.06	−0.14 ± 0.04
% change	−2.4 ± 0.7	−2.2 ± 1.1	−1.9 ± 1.4	−3.1 ± 1.0
TGs,^2^ mmol/L				
Week 0	1.39 ± 0.04	1.37 ± 0.06	1.43 ± 0.09	1.39 ± 0.07
Week 3	1.35 ± 0.04	1.29 ± 0.07	1.33 ± 0.06	1.43 ± 0.07
Week 6	1.35 ± 0.04	1.28 ± 0.06	1.34 ± 0.06	1.42 ± 0.07
Net change	−0.05 ± 0.03	−0.09 ± 0.04	−0.08 ± 0.05	0.03 ± 0.06
% change	4.4 ± 3.1	−1.7 ± 3.4	2.3 ± 3.8	12.8 ± 7.6
Glucose, mmol/L				
Week 0	5.42 ± 0.03	5.37 ± 0.05	5.40 ± 0.06	5.48 ± 0.04
Week 3	5.38 ± 0.03	5.36 ± 0.05	5.33 ± 0.06	5.46 ± 0.05
Week 6	5.39 ± 0.03	5.34 ± 0.05	5.34 ± 0.05	5.47 ± 0.05
Net change	−0.03 ± 0.02	−0.03 ± 0.04	−0.05 ± 0.05	−0.01 ± 0.03
% change	−0.4 ± 0.4	−0.4 ± 0.7	−0.6 ± 0.8	−0.1 ± 0.6
Insulin,^2^ pmol/L				
Week 0	65.0 ± 2.3	63.3 ± 3.8	65.1 ± 4.1	66.6 ± 4.0
Week 3	65.6 ± 2.3	64.6 ± 4.3	63.9 ± 3.8	68.2 ± 3.8
Week 6	62.5 ± 2.1	62.1 ± 3.4	62.5 ± 3.9	63.0 ± 3.9
Net change	−2.5 ± 1.5	−1.2 ± 2.0	−2.6 ± 2.9	−3.5 ± 2.8
% change	3.3 ± 2.5	5.3 ± 4.1	3.9 ± 4.4	0.7 ± 4.7
HOMA-IR^2^				
Week 0	1.27 ± 0.05	1.20 ± 0.07	1.28 ± 0.09	1.33 ± 0.10
Week 3	1.24 ± 0.04	1.21 ± 0.08	1.22 ± 0.08	1.29 ± 0.07
Week 6	1.18 ± 0.04	1.17 ± 0.06	1.18 ± 0.08	1.20 ± 0.07
Net change	−0.09 ± 0.04	−0.03 ± 0.04	−0.10 ± 0.08	−0.14 ± 0.06
% change	2.4 ± 2.7	6.3 ± 4.8	3.6 ± 5.1	−2.6 ± 4.2
CRP,^2^ μg/L				
Week 0	1.95 ± 0.15	1.70 ± 0.18	2.10 ± 0.29	2.04 ± 0.32
Week 3	2.15 ± 0.17	1.82 ± 0.18	2.49 ± 0.36	2.15 ± 0.29
Week 6	2.28 ± 0.17	1.90 ± 0.19	2.60 ± 0.37	2.35 ± 0.31
Net change	0.34 ± 0.12	0.20 ± 0.13	0.50 ± 0.26	0.32 ± 0.20
% change	45.3 ± 8.5	35.5 ± 14.7	52.6 ± 17.2	48.1 ± 12.0

1 Values are group means ± SEMs. The HDS group received 25 g/d soy protein; the LDS group received 12.5 g/d soy protein and 12.5 g/d whey protein; and the control group received 25 g/d whey protein. Net change = (week 6 − week 0) absolute values. % change = [(week 6 – week 0)/wk 0] × 100. The changes in outcome variables after 3 wk (not shown) and 6 wk of treatment did not differ between groups, *P* > 0.05. CRP, C-reactive protein; HDS, high-dose soy; LDS, low-dose soy.

2 Values were transformed with the use of ln (TGs, insulin, and HOMA-IR) and reciprocal transformation (CRP) before analysis.

When week 3 measurements were compared with baseline values, there were no significant differences in outcome variables between the 3 study groups (Table 4). On intention-to-treat analysis, the change in LDL cholesterol after 6 wk of treatment, was not significantly different between the study groups. When expressed as a percentage change from baseline values, the differences between treatments remained nonsignificant. Additionally, at the end of the study, we found no significant difference between treatments for total cholesterol, HDL cholesterol, total-to-HDL cholesterol ratio, TGs, CRP, glucose, insulin, HOMA-IR, blood pressure, or Framingham 10 y CHD risk score (Table 4). As expected, median plasma isoflavone concentrations increased significantly from baseline in the HDS and LDS groups to 347 μg/L and 146 μg/L, respectively, at week 3, and 419 and 119 μg/L, respectively, at week 6—values that were significantly higher than in the control group (1 and 2 μg/L, *P* < 0.001).

ANCOVA showed no confounding effects from age, gender, BMI, center, and change in dietary intake (total energy, protein, isoflavones, saturated fat, and dietary fiber) on change in LDL cholesterol. At the end of the study, change in LDL cholesterol in equol producers (13.4%) was not significantly different from that of equol nonproducers (data not shown). Pooling the soy treatment groups and comparing outcome variables with those of the control group also resulted in no difference by treatment.

## Discussion

We conducted a randomized, controlled, double-blind trial to assess the LDL cholesterol–lowering effect of DWSF incorporated into a muffin and consumed at 2 doses. All enrolled participants (*n* = 243) were entered into an intention-to-treat analysis to assess the intervention effect, which showed that neither dose of DWSF (12.5 or 25 g/d soy protein) had a significant LDL cholesterol lowering effect. Furthermore, there were no significant treatment effects on total- or HDL cholesterol, TGs, CRP, HOMA-IR, blood pressure, or Framingham 10 y CHD risk score. The study muffins were formulated to meet the FDA-recommended intake of soy protein (25 g/d) for cholesterol reduction (11) and, despite excellent compliance, no intervention effects were observed. Few studies, to our knowledge, have examined the dose-dependent relation between soy protein and LDL cholesterol, making it difficult to estimate a minimum effective dose to elicit a cholesterol-lowering response. The majority of human studies on soy have used soy protein powders in doses of between 15 and 50 g/d (19), although a recent trial used 12.8 g soy protein as a beverage supplement (40 g soy flour) for 6 mo in hypercholesterolemic, equol-producing postmenopausal women and achieved a significant reduction (6.32%) in LDL cholesterol (37). This dose was similar to the lower amount used in our study; however, participants in the study by Liu et al. (37) were not representative of the general population, which makes it difficult to compare the 2 studies.

Our finding of a null effect from DWSF on LDL cholesterol is consistent with the 2006 meta-analysis by the AHA Science Advisory, which concluded that current evidence from human intervention studies does not support the claim for a clinically relevant health benefit, and the ∼3% reduction in LDL cholesterol is very small relative to the large amount of soy protein tested in human studies (20). Additionally, a recent review compared key studies used in the FDA soy protein health claim petition with more recent trials and concluded that there were, at best, modest effects from soy protein on the blood lipid profile, even when amounts of soy protein beyond the FDA recommendation of 25 g/d were used (21). However, our results are in contrast to the findings of a meta-analysis by Anderson and Bush (19), who performed a quality assessment of 43 clinical trials published since the approval of the 1999 FDA health claim. These authors concluded that soy protein lowered LDL cholesterol by ∼5%, increased HDL cholesterol by 3.2%, and decreased TGs by 10.7% (19). Similar findings were reported in an analysis by Health Canada (23). Clearly, the reasons for these inconsistencies require further investigation, but they likely could be due to the fact that the majority of parallel controlled human trials have involved small samples of selected groups of participants and mostly used ISP as the intervention vehicle. Many of these studies (reviewed in 20, 21, 23) recruited postmenopausal women or hypercholesterolemic men, which conflicts with one of the requirements for health claim substantiation: that the study population must be representative of the general population. Furthermore, most studies involved between 30 and 70 participants with duration of treatment ranging from 4 to 13 wk, and 8 wk suggested as being optimal (19). It seems probable that soy protein may be of benefit to selected groups in the population when given in the right dose and for ∼8 wk; however, whether this can be translated to the general population is likely to remain a contentious issue.

Health claim assessments should take into consideration the influence of dietary sources on constituent bioactivity. In the case of soy, the majority of studies have used ISP or isolated isoflavones, which seems reasonable, because there is evidence suggesting that the cholesterol-lowering ability of soy is principally exerted by peptides derived from 7S protein (β-conglycinin), which was found to alter lipid metabolism in HepG2 cells (38). Soy protein also may lower serum cholesterol by promoting bile acid excretion, given that it was recently shown to regulate key enzymes involved in reverse cholesterol transport (39). Several other potential mechanisms describing the cholesterol-lowering effect of soy have been suggested (40); however, it is unclear whether bioactive soy peptides are able to enter circulation intact. A study with Caco-2 cells suggests that peptides from β-conglycinin are bioavailable (41). Few studies have tested the bioactivity of soy protein fractions in humans; however, preliminary findings demonstrating antilipidemic effects in hyperlipidemic adults (42, 43) support the role of β-conglycinin as the active ingredient in soy.

DWSF is widely used in food manufacturing and represents a major source of dietary soy. As such, we used a soy muffin as the intervention vehicle because we wanted to assess the LDL cholesterol–lowering efficacy of DWSF as opposed to ISP. Two previous studies that used a combination of ISP and muffins for interventions in postmenopausal women and hypercholesterolemic men (44) and perimenopausal women (44, 45) failed to demonstrate an LDL cholesterol–lowering effect, which is consistent with our findings. It appears that the LDL cholesterol–lowering effect of soy is lost when ISP or DWSF is incorporated into a muffin. Other studies examining the cholesterol-lowering effect of baked soy flour products have produced mixed results. Previously, Ridges et al. (46) observed significant reductions in LDL cholesterol when baked soy products, enriched with a combination of soy flour, soy grits, and linseed, were consumed for 3 wk; however, this effect became nonsignificant at the end of the 8 wk trial (46). Jenkins et al. (47) did not detect a hypolipidemic effect after a cereal made from DWSF was consumed for 3 wk. Ahn-Jarvis et al. (48) found that bread made with a combination of DWSF and soy milk significantly lowered LDL cholesterol in hypercholesterolemic adults, and this was related to total urinary isoflavone concentrations (48). However, recently, Moghaddam et al. (49) showed that bread made from soybean flour did not improve serum lipids in diabetic women. Taken together, these results suggest that food matrix and/or processing, particularly baking at high temperatures, may affect the bioactivity of soy flour.

We recently completed preliminary characterization studies (data not shown), in which gel electrophoresis scans showed that the ratio of 11S:7S globulin in DWSF was typical for the soy flour used in our study, and that the protein subunits are preserved and clearly visible in the baked muffin samples. However, processing into muffins resulted in a decrease in the β-conglycinin (7S) band relative to glycinin (11S). This reduction of β-conglycinin in the extracted protein could possibly account for the loss of LDL cholesterol–lowering ability of baked soy muffins, which was evident in our study.

Another potential issue could be related to the storage of the study muffins: after baking they were stored frozen for several weeks before being given to participants. Although it is unlikely that the muffins would have undergone many freeze/thaw cycles, this has been shown to affect the bioavailability of putative bioactive ingredients (50), which could have influenced the study outcome. Clearly, there is a need to fill the many existing gaps to better understand the effect of processing methods on the lipid-lowering efficacy of soy and its derived products (51). Moreover, there are considerable differences in the way soy foods are prepared in Asia compared with North America and Europe, which may in turn influence the metabolism, absorption, and health benefits of soy bioactive ingredients.

Our results showed that plasma isoflavone concentrations increased in a dose-dependent manner, indicating that the isoflavones from DWSF are bioavailable, but this did not appear to influence blood lipids. It has been suggested that the health benefits of soy may be enhanced in persons with the ability to produce *S*-equol, a metabolic derivative of the isoflavone daidzein (52). Equol is thought to reduce CHD risk factors through its action as a potent antioxidant, and by upregulating genes involved in the production of endothelial nitric oxide synthase (53). In the present study, the intervention effect on LDL cholesterol in equol nonproducers was not significantly different from that in equol producers, and although the sample size for the latter analysis was small (*n* = 19), this is consistent with findings reported elsewhere (53). Interestingly, Wong et al. (54) showed that the hypocholesterolemic effect of soy is potentiated through the addition of a prebiotic, presumably by promoting colonic conversion of the soy isoflavone daidzein to equol, and is associated with an increase in SCFA production (54). The proportion of equol producers in the current study was much lower than expected (13.4%), suggesting an unusual characteristic of the study population examined perhaps relating to differences in gut microbiota or an exaggeration of previous estimates. The latter is supported by a recent study that identified a relatively low proportion of equol producers (17.5%) in postmenopausal women in Birmingham, Alabama (55). It is expected that the role of the gut microbiota in potentiating the LDL cholesterol–lowering efficacy of soy will be further examined in future studies.

In conclusion, we used a double-blind, randomized controlled trial to assess the dose effect of DWSF incorporated into muffins, and found that daily consumption of muffins providing 12.5 g or 25 g/d soy protein did not significantly lower LDL cholesterol in adults with elevated cholesterol. Given the inconsistency in the outcomes from soy human clinical trials, it is imperative that the role of the food matrices and soy bioactive ingredients used for intervention be further examined in the participants who are representative of the general population; this is critical for the advancement of a soy health claim.

## Footnotes

8 Abbreviations used: CHD, coronary heart disease; CRF, case report form; CRP, C-reactive protein; DWSF, defatted whole soy flour; HDS, high-dose soy; ISP, isolated soy protein; LDS, low-dose soy.
